# Virological outcomes and risk factors for non-suppression for routine and repeat viral load testing after enhanced adherence counselling during viral load testing scale-up in Zimbabwe: analytic cross-sectional study using laboratory data from 2014 to 2018

**DOI:** 10.1186/s12981-022-00458-z

**Published:** 2022-07-09

**Authors:** Trudy Tholakele Mhlanga, Bart K. M. Jacobs, Tom Decroo, Emma Govere, Hilda Bara, Prosper Chonzi, Ngwarai Sithole, Tsitsi Apollo, Wim Van Damme, Simbarashe Rusakaniko, Lutgarde Lynen, Richard Makurumidze

**Affiliations:** 1 Institute of Tropical Medicine, Antwerp, Belgium; 2 Faculty of Medicine and Health Sciences, University of Zimbabwe, Harare, Zimbabwe; 3 Gerontology, Faculty of Medicine & Pharmacy, Vrije Universiteit Brussel (VUB), Brussels, Belgium; 4 AIDS & TB Unit, Ministry of Health & Child Care, Harare, Zimbabwe; 5 Research Foundation of Flanders, Brussels, Belgium; 6 Harare City Council, Department of Health, Harare, Zimbabwe

**Keywords:** HIV viral load testing, ART, Viral load non-suppression, Implementation, Zimbabwe

## Abstract

**Background:**

Since the scale-up of routine viral load (VL) testing started in 2016, there is limited evidence on VL suppression rates under programmatic settings and groups at risk of non-suppression. We conducted a study to estimate VL non-suppression (> 1000 copies/ml) and its risk factors using "routine" and "repeat after enhanced adherence counselling (EAC)" VL results.

**Methods:**

We conducted an analytic cross-sectional study using secondary VL testing data collected between 2014 and 2018 from a centrally located laboratory. We analysed data from routine tests and repeat tests after an individual received EAC. Our outcome was viral load non-suppression. Bivariable and multivariable logistic regression was performed to identify factors associated with having VL non-suppression for routine and repeat VL.

**Results:**

We analysed 103,609 VL test results (101,725 routine and 1884 repeat test results) collected from the country’s ten provinces. Of the 101,725 routine and 1884 repeat VL tests, 13.8% and 52.9% were non-suppressed, respectively. Only one in seven (1:7) of the non-suppressed routine VL tests had a repeat test after EAC. For routine VL tests; males (vs females, adjusted odds ratio (aOR) = 1.19, [95% CI 1.14–1.24]) and adolescents (10–19 years) (vs adults (25–49 years), aOR = 3.11, [95% CI 2.9–3.31]) were more at risk of VL non-suppression. The patients who received care at the secondary level (vs primary, aOR = 1.21, [95% CI 1.17–1.26]) and tertiary level (vs primary, aOR = 1.63, [95% CI 1.44–1.85]) had a higher risk of VL non-suppression compared to the primary level. Those that started ART in 2014–2015 (vs < 2010, aOR = 0.83, [95% CI 0.79–0.88]) and from 2016 onwards (vs < 2010, aOR = 0.84, [95% CI 0.79–0.89]) had a lower risk of VL non-suppression. For repeat VL tests; young adults (20–24 years) (vs adults (25–49 years), (aOR) = 3.48, [95% CI 2.16 -5.83]), adolescents (10–19 years) (vs adults (25–49 years), aOR = 2.76, [95% CI 2.11–3.72]) and children (0–9 years) (vs adults (25–49 years), aOR = 1.51, [95% CI 1.03–2.22]) were at risk of VL non-suppression.

**Conclusion:**

Close to 90% suppression in routine VL shows that Zimbabwe is on track to reach the third UNAIDS target. Strategies to improve the identification of clients with high routine VL results for repeating testing after EAC and ART adherence in subpopulations (men, adolescents and young adolescents) at risk of viral non-suppression should be prioritised.

**Supplementary Information:**

The online version contains supplementary material available at 10.1186/s12981-022-00458-z.

## Introduction

The World Health Organisation (WHO) in 2013 recommended routine viral load (VL) monitoring as the preferred approach [1]. Previously, patient monitoring relied on clinical and immunological criteria followed by targeted VL testing. This approach has a low sensitivity and low predictive value in identifying patients with virological failure [2]. In the ESA (Eastern and Southern Africa) region by the end of 2019, progress towards the 90-90-90 targets among all people living with HIV (PLHIV) was 86%, 72% and 65%, respectively [3]. Access to VL testing in ESA remains a challenge [3]. Some barriers to accessing VL testing include the inability to identify eligible clients, sample collection challenges, inefficient transportation systems to and from the laboratory, lack of capacity by VL testing laboratories (lack of staff, equipment, information systems and quality assurance) and failure by health care workers to use the results for decision making and clinical management of patients [4].

Factors associated with non-viral suppression in sub-Saharan Africa among patients on antiretroviral therapy (ART) with access to routine VL testing include being a child, adolescent, male, first-line ART, advanced HIV disease, prior ART exposure, longer duration on ART, among others [5–8]. In a meta-analysis, with mainly African studies, almost half of patients with an initially elevated VL re-suppressed following enhanced adherence counselling (EAC) [9]. Re-suppression was higher among those with enhanced adherence support and lower in children and adolescents. Of those on first-line ART with confirmed virological failure, about half (53.4%) were switched to a different regimen [9]. These gaps highlight the importance of conducting VL cascade analyses in low and middle-income countries, enabling them to address gaps with targeted measures.

In Zimbabwe, Treat All, i.e. ART regardless of the patient's clinical or immunological criteria, was implemented since 2016 [10]. The country started to scale-up routine VL testing in 2016 as 2015 WHO Treat All guidelines were adopted [1]. The VL guidelines recommended "routine VL" to be collected at six and 12 months after ART initiation and then every 12 months [11]. Patients with a VL above 1000 copies/ml are referred for EAC sessions for three months (12 weeks). These sessions aim at identifying and addressing problems causing the high VL. A "repeat VL" test is conducted after 12 weeks of EAC sessions. However, if the patient still has adherence issues, the EAC sessions are extended further for a month before the repeat VL test. If the repeat VL result remains above 1000 copies/ml and adherence proven to be satisfactory during the EACs, the patient is referred to be switched to a second-line regimen. If the repeat VL is below 1000 copies/ml, the patient continues with the first-line treatment regimen [12].

Zimbabwe is among the seven countries in ESA that have met the UNAIDS 90-90-90 targets [3]. According to UNAIDS, at the end of 2019, among all PLHIV, 90% were aware of their HIV status, 85% were on ART, and 77% had virological suppression [3]. These findings have also been confirmed by the recent Zimbabwe Population-Based HIV Impact Assessment (ZIMPHIA) 2020 [13]. Despite the good overall performance towards the 3rd 90; the following challenges across the VL cascade were identified: limited access to VL testing, long turnaround times, patients with high VL not completing EAC sessions, a high proportion of patients not re-suppressing after EAC and or not being switched to second-line ART after virological failure was confirmed [14–19]. The few studies that have been conducted focused on specific regions, subpopulations, tertiary institutions, donor-funded non-governmental organisations/districts or used electronic databases only available at selected sites [14–20].

Furthermore, previous studies used health facility data from patient medical records. Considering the challenges associated with the processes of returning VL results from testing laboratories back to the requesting health facilities for utilisation in patient management, the use of laboratory data might give a better picture of VL testing outcomes. We, therefore, conducted a study using laboratory data from a centralised VL testing laboratory that receives samples from across the country to estimate VL suppression for routine and repeat VL tests, done after EAC, and identified risk factors for non-suppression.

## Methods

### Study design

An analytic cross-sectional study was conducted using routinely collected secondary VL testing laboratory data between 2014 and 2018.

### Study setting

The private sector and non-governmental organisations, which included Médecins Sans Frontiers’, pioneered VL testing in Zimbabwe around 2012 [15]. VL testing targeted those with immunological and/or clinical treatment failure. To achieve the third UNAIDS target, a Viral Load National Scale-up Plan (2015–2018) was developed [21]. This paved the way for the scale-up of routine VL testing in the public sector. The plan aimed at reaching 90% coverage by 2018. However, by the end of 2018, VL testing coverage among patients on ART was 60%, and this varied significantly across different subpopulations and provinces [22]. As part of the VL testing scale-up, testing laboratories were to be established in the country's ten provinces. In 2020, six provinces (Bulawayo, Harare, Mashonaland West, Masvingo, Midlands and Mutare) had VL testing laboratories. Each of the VL testing laboratories has its own laboratory information management system (LIMS) specific to the management of VL data.

During the collection of blood samples for VL testing at health facility level, the clinician complete a VL request form. The VL request form collects demographic, health facility, clinical and EAC sessions information. It also collects the patient mobile number and consent to receive a mobile text message reminder when the results are ready. The VL request form accompanies the specimen to the laboratory for VL testing. After testing, the laboratory enters the information on the VL request form together with VL result in the LIMS. The results are communicated back to the facility via three main methods i.e. email, frontline mobile system and paper print outs.

### Study participants and sampling strategy

At the preliminary stage of the study, we managed to get VL testing data from five of the six provinces. After assessing the quality of data, we purposively selected Beatrice Road Infectious Disease Hospital (BRIDH) because it receives samples from the country's ten provinces and its LIMS had more programmatic and clinically relevant variables with higher completeness. We could not merge the data from the other VL testing laboratories due to different LIMS used which led to different data formats and variables. The study participants were PLHIV on ART, who had a VL test done in Zimbabwe between 2014 and 2018. All VL results produced by BRIDH between 2014 and 2018 were included in the study. We only included VL tests that were either "routine" or "repeat after EAC". We excluded results from targeted VL, which were done in patients with presumed immunological or clinical failure.

### Data sources and study variables

The data were obtained from the LIMS databases at BRIDH. The data were extracted in MS-Excel format and imported into the statistical software for cleaning before analysis. The following exposure variables were extracted from the LIMS database: age, sex, pregnancy status, breastfeeding status, ART start date, ART regimen, level of care, the reason for VL test, attendance of EAC sessions, time on ART for VL testing, VL test results and consent to receive mobile message notification when results are ready. The time on ART for VL testing was calculated as the time between the date of ART initiation and the date of the VL testing specimen collection. The age variable was categorised into the following ages in years; children (0–9), adolescents (10–19), young adults (20–24), adults (25–49) and adults 50 years and above (≥ 50 years). The outcome of our study was VL suppression. A suppressed VL was defined as a VL result of fewer than 1000 copies/ml, and non-suppression was defined by a VL greater than or equal to 1000 copies/ml.

### Data analysis

The data were analysed using the R statistical software version 3.6.1 [23]. The analysis was performed separately for two groups, for routine VL testing and repeat VL testing. We summarised the frequencies and proportions for exposure variables. We performed a trend analysis of VL non-suppression over time on ART by age groups and assessed the significancy of the trends using a generalised additive model (GAM). The association of exposure variables with having a non-suppressed VL was assessed using Pearson's chi-squared test. Bivariable and multivariable logistic regression was performed to identify factors associated with having a non-suppressed VL in each of the two groups. For the multivariable regression, we employed a hierarchical approach. We included in the multivariable model all variables associated with p-value < 0.1 in the univariate analyses. A stepwise backward elimination was used, and all variables having a p-value < 0.05 were retained in the final model.

## Results

### Study participants

**Fig. 1:**
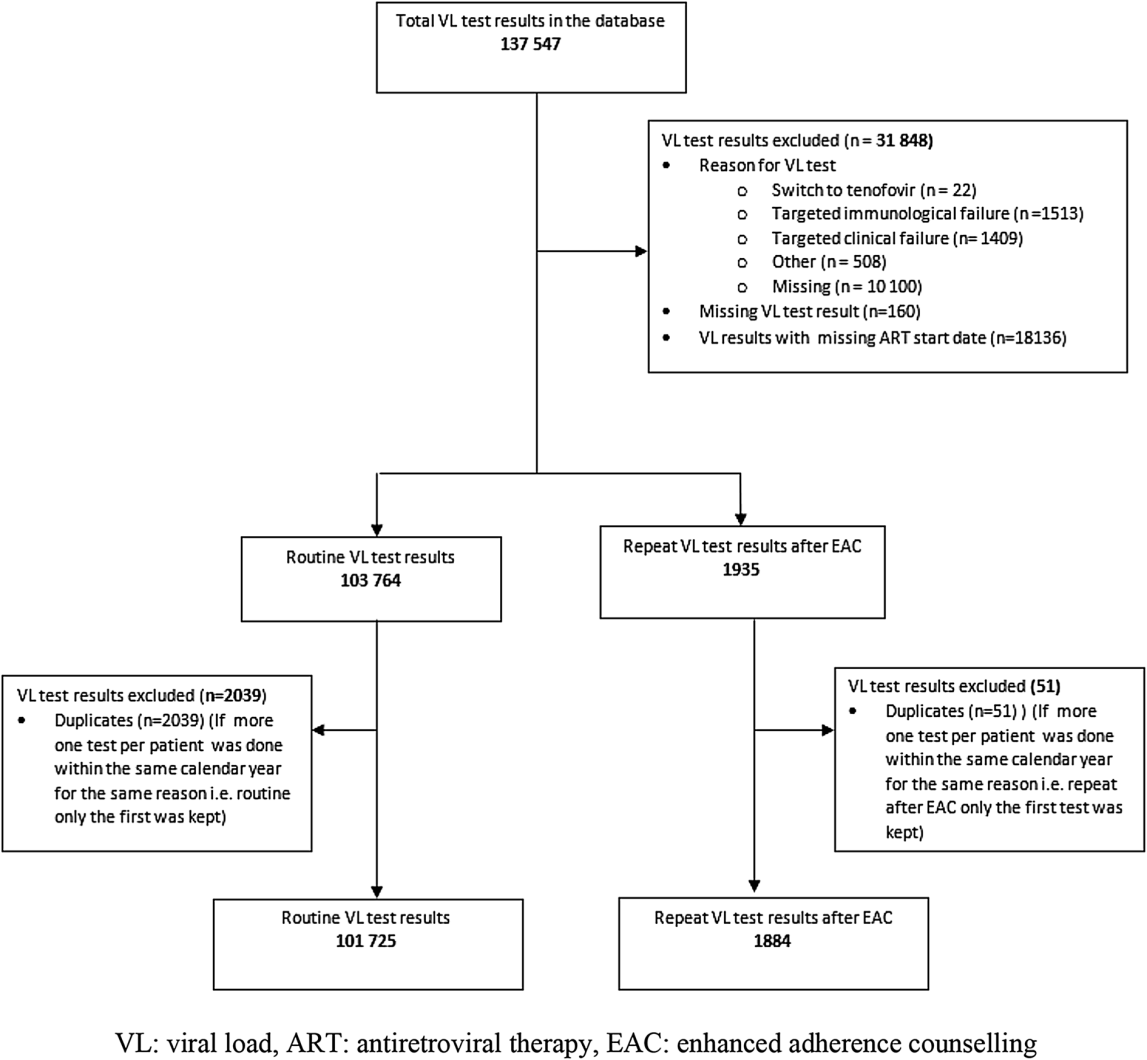
Routine and repeat after enhanced adherence counselling study participants

We analysed 103,609 VL test results (101,725 routine and 1884 repeat after EAC VL test results) conducted between 2014 and 2018 (Fig. 1). The VL test results were from samples collected from the country's ten provinces. Harare Province, in which the BRIDH VL testing laboratory is based, had the highest number of tests conducted (40.3%), followed by neighbouring Mashonaland East (31%) and Masvingo (11.7%).

### Estimation of overall viral load suppression

Routine VL and repeat VL after EAC results were non-suppressed in 13.8% (14,020/101725) and 52.9% (996/1884) of the study participants, respectively (Table 1). From the 14,020 non-suppressed routine VL tests, an estimated 1884 had repeat VL tests and this translate to about one in seven (1:7) of the non-suppressed routine VL tests having a repeat test.

**Table 1:** Viral load suppression for routine and repeat viral load tests done between 2014 and 2018

Variable	Category	Total	Suppression		Non-suppression	
			n	%	n	%
Reason for test	Routine viral load test	101,725	87,705	86.2	14,020	13.8
	Repeat after enhanced adherence counselling viral load test	1884	888	47.1	996	52.9

### Routine viral load test results

#### Patients’ characteristics of routine viral load test results

The total number of routine VL tests included in the analysis was 101 725. The majority were females (61%). The median age in years of the patients was 41 (IQR: 33–48). The majority were adults (25–49 years) (64.3%) followed by 21.2% adults 50 years and above, 5.0% adolescents (10–19 years), 3.4% young adults (20–24 years) and 2.5% children (0–9 years). Patients initiated ART in different periods, with 28.4% starting in 2010 or earlier, 33.7% between 2011 and 2013, 24.5% between 2014 and 2015 and 13.1% in 2016 or later. Most of the patients were on a tenofovir-based non-nucleoside reverse transcriptase inhibitor (TDF based NNRTI) regimen (93%). There were 2.6% confirmed breastfeeding women and 4.5% confirmed pregnant women. Most of the patients received care at the primary level (64.5%) and least at the tertiary level (1.6%) (Table 2).

**Table 2:** Characteristics of patients who had routine viral load tests done between 2014 and 2018

Variable	Category	N = (101,725)	Percentage (%)
Sex	Female	62,040	61.0
	Male	36,915	36.3
	Missing	2770	2.7
Median age IQR (Q1:Q3)		41(33–48)	
Age	Children (0–9 years)	2544	2.5
	Adolescents (10–19 years)	5048	5.0
	Young adults (20–24 years)	3434	3.4
	Adults (25–49 years)	65,449	64.3
	Adults (≥ 50 years)	21,545	21.2
	Missing	3705	3.6
Year of ART initiation	< 2010	29,181	28.7
	2011–2013	34,251	33.7
	2014–2015	24,947	24.5
	> 2016	13,346	13.1
ART regimen	TDF based NNRTI	94,625	93.0
	non-based TDF NNRTI	6759	6.6
	PI Based	305	0.3
	Other	36	< 0.1
Pregnant	Confirmed	2695	2.6
	Unconfirmed*	99,030	97.4
Breastfeeding	Confirmed	4579	4.5
	Unconfirmed*	97,146	95.5
Consent to receive VL results notification via mobile text message reminder	Yes	76,550	75.3
	No	19,297	19.0
	Missing	5878	5.8
Level of care	Primary	65,564	64.5
	Secondary	34,371	33.8
	Tertiary	1657	1.6
	Missing	133	0.1

*IQR* interquartile range, *TDF* tenofovir, *NNRTI* non-nucleoside reverse transcriptase inhibitor, *NRTI* nucleoside reverse transcriptase inhibitor, *PI* protease inhibitor, *VL* viral load

^*^Unconfirmed includes males and females of all ages

#### Viral load non-suppression over time for routine VL test results

**Fig. 2:**
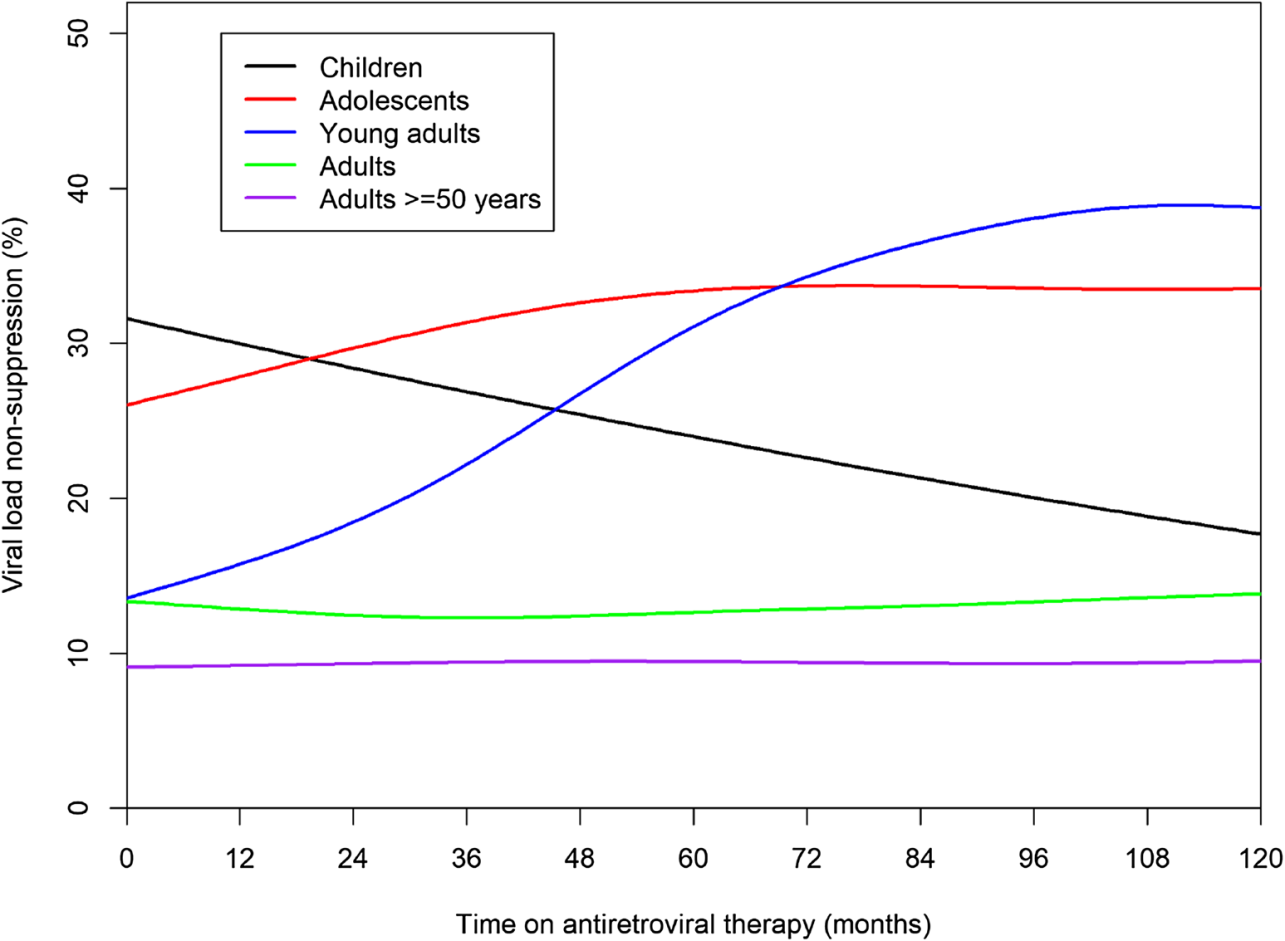
Routine viral load non-suppression over time on ART by age groups between 2014 and 2018

Trend analysis for VL non-suppression by age group was assessed for about 120 months (Fig. 2). In children (0–9 years), routine VL non-suppression decreased overtime on ART and the trend was significant (p-value-0.001). In adolescents (10–19 years) and young adults (20–24 years), VL non-suppression increased with time on ART and both the trend were significant i.e. (p-value- 0.007) and (p-value < 0.001) respectively. In adults (25–49 years) and the adults 50 years and above VL, non-suppression was approximately constant over time on ART with p-value—0.010) and (p-value—0.442) respectively. However, the clinically not meaningful change for adults was found to be significant.

#### Association of exposure variables and viral load non-suppression for routine VL tests

In the bivariate analysis, we found a statistically significant association between all exposure variables and VL non-suppression (p < 0.05). Males had a higher VL non-suppression (15.3%) than females (12.9%). VL non-suppression was the highest in adolescents (10–19 years) (32.8%), followed by children (0–9 years) (26.9%), young adults (20–24 years) (24.3%) and adults 50 years and above (9.8%). According to the year of ART initiation, there were statistically significant but small differences for VL non-suppression ranging from 13.0 to 14.2%. VL non-suppression was 12.4% and 10.9%, respectively, among confirmed breastfeeding and pregnant women. Patients who had a VL test result at the tertiary level had the highest VL non-suppression (20.2%), patients at the primary level had the lowest (12.7%) (Table 3).

**Table 3:** Association between exposure variables and viral load non-suppression for routine tests done between 2014 and 2018

Variable	Category	Suppression		Non-suppression		p-value**
		N	%	N	%	
Sex	Female	54,048	87.1	7992	12.9	** < 0.001**
	Male	31,255	84.7	5660	15.3	
	Missing	2402	86.7	368	13.3	
Age	Children (0–9 years)	1860	73.1	684	26.9	** < 0.001**
	Adolescents (10–19 years)	3393	67.2	1655	32.8	
	Young adults (20–24 years)	2601	75.7	833	24.3	
	Adults (25–49 years)	57,160	87.3	8289	12.7	
	Adults (≥ 50 years)	19,467	90.4	2078	9.8	
Year of ART initiation	< 2010	25,023	85.8	4158	14.2	** < 0.001**
	2011–2013	29,383	85.8	4868	14.2	
	2014–2015	21,693	87.0	3254	13.0	
	> 2016	11,606	87.0	1740	13.0	
Breastfeeding	Confirmed	4010	87.6	569	12.4	**0.006**
	Unconfirmed*	83,695	86.2	13,451	13.8	
Pregnant	Confirmed	2402	89.1	292	10.9	** < 0.001**
	Unconfirmed*	85,303	86.1	13,727	13.9	
Consent to receive VL results notification via mobile text message reminder	Yes	66,394	86.7	10,156	13.3	** < 0.001**
	No	16,322	84.6	2975	15.4	
	Missing	4989	84.9	889	15.1	
Level of care	Primary	57,244	87.3	8320	12.7	** < 0.001**
	Secondary	29,024	84.4	5347	15.6	
	Tertiary	1322	79.8	335	20.2	
	Missing	115	86.5	18	13.5	

The bold is to emphasis statistically significant p-values

^*^Unconfirmed includes males and females of all ages

^**^Chi-square

#### Risk factors for non-suppression for routine viral load tests

After adjusting for potential confounders, sex, pregnancy, age, year of ART initiation, the level of care and consent to receive a text message was associated with VL non-suppression. Males (vs females, adjusted odds ratio (aOR) = 1.19, [95% CI 1.14 −1.24]) were at an increased risk of VL non-suppression. Adolescents (10–19 years) (vs adults (25–49 years), aOR = 3.11, [95% CI 2.91- 3.31]) were considerably more at risk of VL non-suppression, followed by children (0–9 years) (vs adults (25–49 years), aOR = 2.40, [95% CI 2.19–2.62]) and young adults (20–24 years) (vs adults (25–49 years), aOR = 2.26, [95% CI 2.08–2.46]. Adults 50 years above (vs adults (25–49 years), aOR = 0.68, [95% CI 0.68–0.72]) had the least risk of VL non-suppression. Those who started ART later, had a significantly lower risk of VL non-suppression. While the difference was small in 2011–2013 (vs < 2010, aOR = 0.94, [0.90–0.99]), it was more substantial in 2014–2015 (vs < 2010, aOR = 0.83, [0.79–0.88]) and from 2016 onwards (vs < 2010, aOR = 0.84, [0.79–0.89]). Pregnant women (vs women without pregnancy data, aOR = 0.79, [95% CI 0.70- 0.90]) had a 20% lower risk of non-suppression than women without confirmed pregnancy. The risk of VL non-suppression increased with the level of care. The patients who received care at the secondary level (vs primary, aOR = 1.21, [95% CI 1.17–1.26]) and tertiary level (vs primary, aOR = 1.63, [95% CI 1.44–1.85]) had a higher risk of VL non-suppression compared to the primary level (Table 4).

**Table 4:** Bivariate and multivariate logistic regression for routine viral load tests done between 2014 and 2018

Variables	Categories	OR	p-value	(95% CI)	aOR	p-value	(95% CI)
Sex	Female	1	** < 0.001**			** < 0.001**	
	Male	1.22		(1.18–1.27)	1.19		(1.14–1.24)
	Missing	1.04		(0.90–1.16)	1.01		(0.90–1.14)
Age	Adults	1	** < 0.001**			** < 0.001**	
	Children (0–9 years)	2.54		(2.31–2.77)	2.40		(2.19–2.62)
	Adolescents (10–19 years)	3.36		(3.16–3.58)	3.11		(2.91–3.31)
	Young adults (20–24 years)	2.21		(2.03–2.39)	2.26		(2.08–2.46)
	Adults (≥ 50 years)	0.74		(0.70–0.77)	0.68		(0.64–0.72)
Year of ART initiation	< 2010	1	** < 0.001**			** < 0.001**	
	2011–2013	1.00		(0.95–1.04)	0.94		(0.90–0.99)
	2014–2015	0.90		(0.86–0.95)	0.83		(0.79–0.88)
	> 2016	0.90		(0.85–0.96)	0.84		(0.79–0.89)
Pregnant	Unconfirmed*	1	** < 0.001**			**0.003**	
	Confirmed	0.76		(0.67–0.86)	0.79		(0.70–0.90)
Consent to receive VL results notification via mobile text message reminder	No	1	** < 0.001**			** < 0.001**	
	Yes	0.84		(0.80–0.88)	0.90		(0.86–0.95)
	Missing	0.98		(0.90–1.06)	0.98		(0.90–1.06)
Breastfeeding	Unconfirmed*	1	**0.006**				
	Confirmed	0.88		(0.81–0.96)			
Level of care	Primary	1	** < 0.001**			** < 0.001**	
	Secondary	1.27		(1.22–1.31)	1.21		(1.17–1.26)
	Tertiary	1.74		(1.54–1.96)	1.63		(1.44–1.85)
	Missing	1.08		(0.63–1.72)	0.96		(0.56–1.56)

The bold is to emphasis statistically significant p-values

^*^Unconfirmed includes males and females of all ages

### Repeat viral load test results after enhanced adherence counselling

We analysed 1884 repeat VL tests after EAC sessions. More females (60.6%) than males had a repeat VL. The median age in years was 37 (IQR: 23–46). There were 54.8% adults (24–49 years), 17.2% adults 50 years and above, 14.3% adolescents (10–19 years), 6.4% children (0–9 years) and 5% young adults (20–24 years). Most of the patients were on a TDF-based NNRTI regimen (81.7%). There were 4.2% breastfeeding women and 1.3% pregnant women. The percentage of patients who consented to receive a mobile text message reminder when results were available was 70.3%. Most of the patients accessed care at the primary level (68.8%) and least at the tertiary level (0.8%). Information on whether EAC sessions were performed on patients with repeat VL tests was missing in 57.4%. Among those with data, 9.9% had at least one EAC session. Furthermore, among those who had EAC sessions performed less than half (48.1%) had three sessions (Additional file 1: Table S1).

On bivariate analysis, sex, age, pregnancy and consent to receive a mobile text message reminder were significantly associated with VL non-suppression (p < 0.05). VL non-suppression (57.4%) was higher in males than in females (50.5%). VL non-suppression was the highest (76.6%) in young adults (20–24 years), followed by adolescents (10–19 years) (72.6%), children (0–9 years) (59.2%), adults (25–49 years) (48.5%) and the lowest in the adults 50 years and above (39.5%). VL non-suppression in women with confirmed pregnancy was 32%. The patients who did not consent to receive a mobile text message reminder had a higher rate of VL non-suppression (59.7%) than those who agreed (Additional file S2: Table S2).

After adjusting for potential confounders, age was associated with VL non-suppression. Young adults (20-24 years) (vs adults (25–49 years), adjusted odds ratio (aOR) = 3.48, [95% CI 2.16 -5.83]), adolescents (10–19 years (vs adults (25–49 years), aOR = 2.76, [95% CI 2.11- 3.72]) and children (0–9 years) (vs adults (25–49 years), aOR = 1.51, [1.03–2.22]) were at higher risk of VL non-suppression. Of the different age groups, the adults 50 years and above (vs adults (25–49 years), aOR = 0.68, [0.53–0.88]) had the lowest risk of VL non-suppression. Pregnant women (vs women without pregnancy data and men, aOR = 0.44, [95% CI 0.18- 1.00]) were at a lower risk of VL non-suppression (Additional file 3: Table S3).

## Discussion

VL non- suppression was 13.8% for routine VL testing and 52.9% for repeat VL testing after EAC. About one in seven (1:7) of the non-suppressed routine VL tests had a repeat test. For routine VL tests, males, adolescents, children, young adults, patients who received care at the secondary and tertiary level of care were at a higher risk of VL non-suppression. Patients who were initiated on ART after 2014 and women with a confirmed pregnancy were at low risk of VL non-suppression. For repeat VL tests, children, adolescents, and young adults had an increased risk of VL non-suppression.

We found routine VL non-suppression of 13.8% (86.2% VL suppression) among patients on ART under programmatic conditions. Our study period preceded the most recent population-based HIV impact assessment survey (November 2019 and March 2020) which reported VL suppression among patient on ART of 90.3% [13]. The prior population-based HIV impact assessment survey had shown VL suppression among patients on ART of 85.3% [24]. If the progress is maintained the country should be on track towards the achievement of the UNAIDS 95–95-95 targets by 2030. The good performance can be attributed to the efforts towards the implementation of the VL Scale-up Plan (2015–2018). The scale-up plan is a multi-sectoral plan that supported decentralised VL testing, increased testing modalities, trained healthcare workers in the collection and utilisation of VL test results [21]. However, the access to VL testing remains low. By the end of 2018, VL testing coverage was approximately 60% and varied across provinces between 20 and 60% [25]. Furthermore, VL testing coverage also varied within the subpopulations, with lower testing among males, children and adolescents [22]. The VL Scale-up Plan experienced funding gaps that have been made worse by the prevailing economic and political challenges the country has been facing [26–28].

Repeat VL non-suppression was 52.9%. This finding is within the range shown by other studies from Zimbabwe and other low resource settings [14, 15, 29, 30]. The VL non-suppression after the EAC sessions is possibly due to the development of HIV drug resistance. However, it is difficult to determine with certainty whether this was due to drug resistance without performing drug resistance testing. Drug resistance testing remains a challenge in most low resource settings, including Zimbabwe [9]. Another study done in Zimbabwe showed a much higher VL non-suppression (83%) among patients with repeat VL testing after targeted VL testing [15]. A higher proportion of non-suppression after targeted testing is to be expected as these patients present with presumed treatment failure based on clinical or immunological criteria.

Our study showed that most patients with high routine VL tests did not get a repeat VL test, with only one in seven (1:7) of patients non-suppressed routine VL tests having a repeat test. Cohort studies in Zimbabwe have also shown that patients with high VL were not getting repeat VL testing. [14–16, 31]. Patients might not be getting repeat VL testing since the filing system for patient medical records is manual and often not well organised. When the VL results are received from the laboratory, healthcare workers do not timeously enter them into patient medical records. VL test results may be lost. There are also laboratory-level challenges that result in a long turnaround time [14, 16]. Point-of-care technology and decentralisation of VL testing are the way forward.

Males were found to be more at risk of VL non-suppression than females. Studies have consistently shown that men are at risk of VL non-suppression [14, 15, 32]. Men might be at risk of VL non-suppression due to poor adherence and later drug resistance caused by their poor health-seeking behaviour leading to non-attendance of clinic appointments to collect medication, work-related commitments, and alcohol use [33–36]. Other health system issues that can contribute to poor adherence to ART among men include inflexible opening hours of clinics not being favourable to the nature of their work and clinics not being male-friendly [37].

We found that patients receiving care at secondary and tertiary levels were at higher risk of VL non-suppression than those receiving care at primary health facilities. Possible explanations for this are the differences in service delivery and organisation of care at the levels. At higher levels of care, follow-up and retention of patients might be poor, which in the long run will affect ART adherence leading to viral non-suppression [38]. Moreover, at higher-level facilities, provision of services is fragmented and specialised [39]. There is constant staff rotation among the nursing staff to fill in staff shortages in other departments [40–42]. They attend to complicated cases, which include suspected cases of treatment failure referred from the lower-level health facilities. In Zimbabwe, confirmation of treatment failure and later switch to second-line therapy is done by medical doctors who are stationed at higher levels of care [39]. Primary level health care facilities are more accessible, less congested, have limited out-of-pocket health expenditure and they offer more differentiated ART service delivery models which include the community ART refill groups [43–45]

Our findings showed that adolescents were at a higher risk of VL non-suppression for routine and repeat after enhanced adherence counselling. Multiple studies have shown that adolescents are at risk of VL non-suppression [20, 29–31, 46–52]. Several reasons, including a lack of psychosocial support, non-disclosure of status, stigma, school-related activities, dependence on a caregiver, non-biological caregiver, and forgetfulness, have been reported as risk factors [46, 52, 53]. In Zimbabwe, we are faced with limited infrastructure and space challenges, and adolescent services are often offered with adults and not separately. This compromises their privacy and makes it uncomfortable to divulge sensitive information [54]. There has also been a high turnover of peer supporter volunteers (Community Adolescent Treatment Supporters) and healthcare workers trained to provide adolescent-friendly services. This has led to adolescent ART, sexual and reproductive health services being handled by cadres that are not trained in dealing with those issues.

We found children at higher risk of VL non-suppression. Some issues, which affect adolescents highlighted above also affect children. However, children, specific issues include limited availability of paediatric fixed-dose ARV and palatable formulations [55]. Our study's interesting finding was that VL non-suppression for children decreased over time. The decrease in VL non-suppression may be explained by the availability of palatable and fixed-dose ARV formulations for older children, gradual independence from caregivers on adherence and possibly the disclosure policy in the country [56]. Disclosure starts as early as six years (partial disclosure) and full disclosure is expected by the age of ten (start of adolescence) [56]. Evidence has shown that both children and adolescents aware of their HIV status are more likely to adhere to treatment and hence have better clinical and virological outcomes [49]. However, the decrease in VL non-suppression over time is affected by several factors such as the children's actual time on ART, the age of starting ART and survivorship bias [57].

In our study we found patients who started ART in recent years, i.e. after 2014 going forward, having a lower risk of viral non-suppression. This can be explained by the gradual adaptation of the WHO HIV treatment guidelines by the country towards starting healthier patients on ART, i.e. Treat All [1]. Few studies to date have shown that patients starting ART under "Treat All" have a better chance of viral load suppression [58–60]. Patients starting ART under "Treat All" are often healthier and have higher CD4 + counts, hence more likely to have better outcomes, including virological suppression [61, 62]. However, during earlier years, i.e. before 2016, most VL tests were based on the targeted approach and were from patients who were failing treatment either clinically or immunologically. There is a possibility that these were documented as routine VL tests and yet they were based on the targeted algorithm.

### Strengths and limitations of the study

Our study was representative, all ten provinces of the country were represented, and we had data from rural and urban settings. The study was also inclusive; it included all age groups (children, adolescents and adults) and pregnant women, a key subgroup. The sample size for routine VL testing was large; it had routine laboratory data collected over 4-years (2014–2018), hence more likely to give robust and precise estimates. The study used routinely collected laboratory data, which can reflect the actual progress of the VL monitoring programme. Overall, the study provided the required snapshot information required by policymakers and stakeholders to optimise the performance of the VL programme.

However, our study had weaknesses and limitations. VL test results from Harare City (urban setting) were overrepresented in the data due to the location of the laboratory in Harare. The patients in the city tend to have better access to health services than those in rural areas. The overrepresentation of patients from Harare might have resulted in the overestimation of non-suppression rates. We were unable to perform a cohort analysis to fully understand the VL performance in the country because most patients in the database had only one VL test result. It was difficult to assess the total number of PLHIV and the total number on ART under the catchment of the BRIDH VL testing laboratory because most of the facilities do not solely send their VL specimens for testing to BRIDH but also other VL testing laboratories in the country. There was also the possibility of under-reporting of the repeat VL tests done as some specimens could have been sent to a different laboratory following the expansion and decentralisation of VL testing. We could not determine the proportion with high repeat VL after EAC switched to second-line therapy because this information is not captured into the LIMS. We intended to analyse the relationship between VL non-suppression with the number of completed EAC sessions but due to the high percentage of missing information (57.4%), we were unable to do so. The information on EAC sessions could have been missing mainly because is not consistently captured in the patient medical records and the clinicians at the time of VL sample collection are unlikely to find it due to work pressure. Our sample size for patients who were tested for routine VL monitoring was very large, and the power of finding associations and statistical significance was high.

Virological suppression cannot be achieved if ART is not taken as prescribed. Hence, adherence to ART is of major importance to prevent the associated public health implications while achieving the specified targets. The findings from this study have shown that the VL monitoring programme has made great strides towards achieving the UNAIDS third 95 target. To ensure that the progress is maintained, several challenges must be addressed. There is a need to put systems in place to identify clients with high routine VL due to repeated testing after EAC sessions. One such system which needs to be optimized and fully implemented is the flagging system with colour codes stickers (red for VL greater or equal to 1000 copies/ml and green for less than 1000 copies/ml) [63]. There is a need to implement targeted interventions to improve adherence to ART among sub-populations at risk of non-viral suppression, especially adolescents and men. For adolescents, home-based support systems and the use of peer community treatment supporters significantly improves adherence and viral suppression [64]. For men, multi-month dispensing, support to those with alcohol and drug use problems can improve adherence to ART. Efforts should be made to make the existing electronic databases, including the LIMS and ePMS interoperable so that data can be consolidated for programming and research purposes.

## Conclusions

In conclusion, our study showed good progress towards achieving the UNAIDS third target in Zimbabwe. However, there is a need to improve the performance of other elements of the viral load cascade, especially the identification of patients with high routine VL results for repeat VL testing after EAC sessions. Attention should also be given to some subgroups which include men, children, adolescents and patients receiving care at higher levels found to be at risk of viral non-suppression.

## Supplementary Information

**Additional file 1**: **Table S1.** Characteristic of patients who had repeat viral load tests between 2014 and 2015. **Table S2.** Association of exposure variables and viral load non-suppression for repeat tests between 2014 and 2018. **Table S3.** Bivariate and multivariate logistic regression analysis for repeat viral load tests done between 2014 and 2018.

## Data Availability

The study was conducted with routinely collected viral load testing programme data of the Zimbabwe National ART Programme available in the Laboratory Information Management System (LIMS). Anyone interested in using the data for scientific or academic purposes should contact the Director of the AIDS and TB Programme, Ministry of Health and Child Care, Government of Zimbabwe, 2nd Floor, Mukwati Building, Harare, Zimbabwe. Email: atp.director@ymail.com.

## Abbreviations

WHO: World Health Organisation
ESA: Eastern and Southern Africa
VL: Viral load
HIV: Human immunodeficiency virus
AIDS: Acquired immunodeficiency syndrome
ART: Antiretroviral therapy
ARV: Antiretroviral drugs
PLHIV: People living with HIV
UNAIDS: Joint United Nations Programme on HIV/AIDS
ZIMPHIA: Zimbabwe Population-based HIV Impact Assessment
BRIDH: Beatrice Road Infectious Disease Hospital
EAC: Enhanced adherence counselling
LIMS: Laboratory information management system
ePMS: Electronic patient management system
CI: Confidence interval
OR: Odds ratio
aOR: Adjusted odds ratio
IQR: Interquartile range
TDF: Tenofovir
NNRTI: Non-nucleoside reverse transcriptase inhibitor
NRTI: Nucleoside reverse transcriptase inhibitor
PI: Protease inhibitor

## References

[1] World Health Organization (WHO) (2016). Consolidated guidelines on the use of antiretroviral drugs for treating and preventing HIV infection: recommendations for a public health approach.

[2] Rutherford GW, Anglemyer A, Easterbrook PJ, Horvath T, Vitoria M, Penazzato M (2014). Predicting treatment failure in adults and children on antiretroviral therapy. AIDS.

[3] United Nations Joint Programme on HIV/AIDS (UNAIDS). UNAIDS Data 2020. Geneva, Switzerland; 2020. https://www.unaids.org/sites/default/files/media_asset/2020_aids-data-book_en.pdf.

[4] El-Sadr WM, Rabkin M, Nkengasong J, Birx DL (2017). Realizing the potential of routine viral load testing in sub-Saharan Africa. J Int AIDS Soc.

[5] Bulage L, Ssewanyana I, Nankabirwa V, Nsubuga F, Kihembo C, Pande G (2017). Factors associated with virological non-suppression among HIV-positive patients on antiretroviral therapy in Uganda, August 2014–July 2015. BMC Infect Dis.

[6] Desta AA, Woldearegay TW, Futwi N, Gebrehiwot GT, Gebru GG, Berhe AA (2020). HIV virological non-suppression and factors associated with non-suppression among adolescents and adults on antiretroviral therapy in northern Ethiopia: a retrospective study. BMC Infect Dis.

[7] Joseph Davey D, Abrahams Z, Feinberg M, Prins M, Serrao C, Medeossi B (2018). Factors associated with recent unsuppressed viral load in HIV-1-infected patients in care on first-line antiretroviral therapy in South Africa. Int J STD AIDS.

[8] Mungwira RG, Divala TH, Nyirenda OM, Kanjala M, Muwalo F, Mkandawire FA (2018). A targeted approach for routine viral load monitoring in Malawian adults on antiretroviral therapy. Trop Med Int Heal.

[9] Ford N, Orrell C, Shubber Z, Apollo T, Vojnov L (2019). HIV viral resuppression following an elevated viral load: a systematic review and meta-analysis. J Int AIDS Soc.

[10] Makurumidze R (2021). Experiences and lessons learnt from the HIV treat all pilot phase implementation in Zimbabwe. HIV/AIDS Res Palliat Care.

[11] World Health Organization (WHO). What’s New in Treatment Monitoring: Viral Load and CD4 Testing. Geneva, Switzerland: WHO; 2017. https://apps.who.int/iris/rest/bitstreams/1086623/retrieve.

[12] Ministry of Health and Child Care (MoHCC). Guidelines for antiretroviral therapy for the prevention and treatment of HIV in Zimbabwe. Harare, Zimbabwe; 2016. https://depts.washington.edu/edgh/zw/vl/project-resources/ZIM_ART_Guidelines_2016_-_review_final.pdf.

[13] ICAP at Columbia University. Zimbabwe Population-based HIV Impact Assessment (ZIMPHIA 2020). Harare, Zimbabwe; 2020. https://phia.icap.columbia.edu/wp-content/uploads/2020/11/ZIMPHIA-2020-Summary-Sheet_Web.pdf.

[14] Bvochora T, Satyanarayana S, Takarinda KC, Bara H, Chonzi P, Komtenza B (2019). Enhanced adherence counselling and viral load suppression in HIV seropositive patients with an initial high viral load in Harare, Zimbabwe: operational issues. PLoS ONE.

[15] Nyagadza B, Kudya N, Mbofana E, Masaka S, Garone D, Chen C-Y (2019). Scaling up HIV viral load monitoring in Manicaland, Zimbabwe: challenges and opportunities from the field. Public Heal Action.

[16] Nyakura J, Shewade HD, Ade S, Mushavi A, Mukungunugwa SH, Chimwaza A (2019). Viral load testing among women on ‘option B+’ in Mazowe, Zimbabwe: how well are we doing?. PLoS ONE.

[17] Apollo T, Takarinda KC, Phillips A, Ndhlovu C, Cowan FM (2021). Provision of HIV viral load testing services in Zimbabwe: secondary data analyses using data from health facilities using the electronic patient monitoring system. PLoS ONE.

[18] Moyo S, Ncube RT, Shewade HD, Ngwenya S, Ndebele W, Takarinda KC (2020). Children and adolescents on anti-retroviral therapy in Bulawayo, Zimbabwe: How many are virally suppressed by month six?. F1000Research.

[19] Shamu T, Chimbetete C, Shawarira-Bote S, Mudzviti T, Luthy R (2017). Outcomes of an HIV cohort after a decade of comprehensive care at Newlands Clinic in Harare, Zimbabwe: TENART cohort. PLoS ONE.

[20] Sithole Z, Mbizvo E, Chonzi P, Mungati M, Juru TP, Shambira G (2018). Virological failure among adolescents on ART, Harare City, 2017—a case-control study. BMC Infect Dis.

[21] Ministry of Health and Child Care Zimbabwe (MoHCC) (2015). Zimbabwe HIV viral load scale-up plan 2015–2018. First.

[22] President’s Emergency Plan for AIDS Relief (PEPFAR). Zimbabwe Country Operational Plan 2019, Strategic Direction Summary. United States of America, Washington; 2019. http://www.pepfar.gov/documents/organization/250290.pdf.

[23] R Core Team (2021). R: A language and environment for statistical computing. R Foundation for Statistical Computing, Vienna, Austria. https://www.R-project.org.

[24] Ministry of Health and Child Care Zimbabwe (MoHCC). Zimbabwe Population-Based HIV Impact Assessment (ZIMPHIA) 2015–2016: Final Report. Harare, Zimbabwe; 2019. https://phia.icap.columbia.edu/wp-content/uploads/2019/08/ZIMPHIA-Final-Report_integrated_Web-1.pdf.

[25] United States of America President’s Emergency Plan for AIDS Relief (PEPFAR). Country Operational Plan 2019, Strategic Direction Summary. United States of America, Washington; 2019. http://www.pepfar.gov/documents/organization/250290.pdf.

[26] Price-Smith AT, Daly JL. HIV/AIDS, state capacity, and political conflict in Zimbabwe. United States of America, Washington: Peaceworks; 2004 (53). https://www.usip.org/sites/default/files/pwks53.pdf.

[27] Mhazo AT, Maponga CC (2022). The political economy of health financing reforms in Zimbabwe: a scoping review. Int J Equity Health.

[28] Jovonna R. Aids in Zimbabwe : how sociopolitical issues hinder the fight against HIV/AIDS. Emory Univ. 2005;1. http://history.emory.edu/home/documents/endeavors/volume1/Jovonnas.pdf.

[29] Laxmeshwar C, Acharya S, Das M, Keskar P, Pazare A, Ingole N (2020). Routine viral load monitoring and enhanced adherence counselling at a public ART centre in Mumbai India. PLoS ONE.

[30] Diress G, Dagne S, Alemnew B, Adane S, Addisu A (2020). Viral load suppression after enhanced adherence counseling and its predictors among high viral load HIV seropositive people in North Wollo zone public hospitals, Northeast Ethiopia, 2019: retrospective cohort study. AIDS Res Treat.

[31] Moyo S, Ncube RT, Shewade HD, Ngwenya S, Ndebele W, Takarinda KC (2020). Children and adolescents on anti-retroviral therapy in Bulawayo, Zimbabwe: how many are virally suppressed by month six?. F1000Research.

[32] Ndagijimana Ntwali JDD, Decroo T, Ribakare M, Kiromera A, Mugwaneza P, Nsanzimana S (2019). Viral load detection and management on first line ART in rural Rwanda. BMC Infect Dis.

[33] Olanrewaju FO, Ajayi LA, Loromeke E, Olanrewaju A, Allo T, Nwannebuife O (2019). Masculinity and men’s health-seeking behaviour in Nigerian academia. Cogent Soc Sci.

[34] Galdas PM, Cheater F, Marshall P (2005). Men and health help-seeking behaviour: literature review. J Adv Nurs.

[35] Gesesew HA, Ward P, Hajito KW, Feyissa GT, Mohammadi L, Mwanri L (2017). Discontinuation from antiretroviral therapy: a continuing challenge among adults in HIV care in ethiopia: a systematic review and meta-analysis. PLoS ONE.

[36] Bukenya D, Mayanja BN, Nakamanya S, Muhumuza R, Seeley J (2019). What causes non-adherence among some individuals on long term antiretroviral therapy? Experiences of individuals with poor viral suppression in Uganda. AIDS Res Ther.

[37] Mantell JE, Masvawure TB, Mapingure M, Apollo T, Gwanzura C, Block L (2019). Engaging men in HIV programmes: a qualitative study of male engagement in community-based antiretroviral refill groups in Zimbabwe. J Int AIDS Soc.

[38] Decroo T, Panunzi I, das Dores C, Maldonado F, Biot M, Ford N,  (2009). Lessons learned during down referral of antiretroviral treatment in Tete Mozambique. J Int AIDS Soc.

[39] McHugh G, Brunskill A, Dauya E, Bandason T, Bwakura T, Duri C (2020). A comparison of HIV outpatient care in primary and secondary healthcare-level settings in Zimbabwe. Public Heal Action.

[40] Kasu TI, Mungure S, Menelik G, Mharakurwa S. The interactions of public health organisational leadership with its environment: a case study of the Parirenyatwa group of hospitals in Harare, Zimbabwe. East Afr Med J. 2021;98. https://www.ajol.info/index.php/eamj/article/view/213025.

[41] Kasu TI, Mungure S, Menelik G, Mharakurwa S. The interactions of public health organisational leadership with its environment: a case study of the Sally Mugabe central hospital in Harare, Zimbabwe. Med J Zambia. 2021;48:85–93. https://www.ajol.info/index.php/mjz/article/view/212948.

[42] Kasu TI, Mungure S, Menelik G, Mharakurwa S. The interactions of public health organisational leadership with its environment: a case study of the Chitungwiza central hospital in Harare, Zimbabwe. East Afr Med J. 2021;98. https://www.ajol.info/index.php/eamj/article/view/224529.

[43] Starfield B, Shi L, Macinko J (2005). Contribution of primary care to health systems and health. Milbank Q.

[44] Bochner AF, Meacham E, Mhungu N, Manyanga P, Petracca F, Muserere C (2019). The rollout of community ART refill groups in Zimbabwe: a qualitative evaluation. J Int AIDS Soc.

[45] Hansen K (2000). The costs of HIV/AIDS care at government hospitals in Zimbabwe. Health Policy Plan.

[46] Nasuuna E, Kigozi J, Babirye L, Muganzi A, Sewankambo NK, Nakanjako D (2018). Low HIV viral suppression rates following the intensive adherence counseling (IAC) program for children and adolescents with viral failure in public health facilities in Uganda. BMC Public Health.

[47] Arpadi SM, Shiau S, De Gusmao EP, Violari A (2017). Routine viral load monitoring in HIV-infected infants and children in low- and middle-income countries: challenges and opportunities. J Int AIDS Soc.

[48] Chhim K, Mburu G, Tuot S, Sopha R, Khol V, Chhoun P (2018). Factors associated with viral non-suppression among adolescents living with HIV in Cambodia: a cross-sectional study. AIDS Res Ther.

[49] Jobanputra K, Parker LA, Azih C, Okello V, Maphalala G, Kershberger B (2015). Factors associated with virological failure and suppression after enhanced adherence counselling, in children, adolescents and adults on antiretroviral therapy for HIV in Swaziland. PLoS ONE.

[50] Kadima J, Patterson E, Mburu M, Blat C, Nyanduko M, Bukusi EA (2018). Adoption of routine virologic testing and predictors of virologic failure among HIV-infected children on antiretroviral treatment in western Kenya. PLoS ONE.

[51] Marcus R, Ferrand RA, Kranzer K, Bekker L-G (2017). The case for viral load testing in adolescents in resource-limited settings. J Int AIDS Soc.

[52] Mavhu W, Berwick J, Chirawu P, Makamba M, Copas A, Dirawo J (2013). Enhancing psychosocial support for HIV positive adolescents in Harare Zimbabwe. PLoS ONE.

[53] Dziva Chikwari C, Ferrand RA, Simms V (2017). Association between self-reported adherence and HIV viral load suppression among older children and adolescents. JAIDS J Acquir Immune Defic Syndr.

[54] Ministry of Health and Child Care Zimbabwe (MoHCC) (2016). National adolescent and youth sexual reproductive health strategy II: 2016–2020.

[55] Waning B, Diedrichsen E, Jambert E, Bärnighausen T, Li Y, Pouw M (2010). The global pediatric antiretroviral market: analyses of product availability and utilization reveal challenges for development of pediatric formulations and HIV/AIDS treatment in children. BMC Pediatr.

[56] Ministry of Health and Child Care Zimbabwe (MoHCC). Zimbabwe national guidelines on HIV testing and counselling. Harare: AIDS and TB directorate; 2014. https://depts.washington.edu/edgh/zw/hit/web/project-resources/HTC_guidelines_children2014.pdf.

[57] Brady MT, Oleske JM, Williams PL, Elgie C, Mofenson LM, Dankner WM (2010). Declines in mortality rates and changes in causes of death in HIV-1-infected children during the HAART Era. JAIDS J Acquir Immune Defic Syndr.

[58] Kerschberger B, Schomaker M, Jobanputra K, Kabore SM, Teck R, Mabhena E (2020). HIV programmatic outcomes following implementation of the ‘Treat-All’ policy in a public sector setting in Eswatini: a prospective cohort study. J Int AIDS Soc.

[59] Hirasen K, Fox MP, Hendrickson CJ, Sineke T, Onoya D (2020). HIV treatment outcomes among patients initiated on antiretroviral therapy pre and post-universal test and treat guidelines in South Africa. Ther Clin Risk Manag.

[60] Dorward J, Sookrajh Y, Gate K, Khubone T, Mtshaka N, Mlisana K (2020). HIV treatment outcomes among people with initiation CD4 counts >500 cells/µL after implementation of treat all in South African public clinics: a retrospective cohort study. J Int AIDS Soc.

[61] Insight Start Study Group (2015). Initiation of antiretroviral therapy in early asymptomatic HIV infection. N Engl J Med.

[62] Groupee TTA 12136 S (2015). A trial of early antiretrovirals and isoniazid preventive therapy in Africa. N Engl J Med.

[63] Ministry of Health and Child Care Zimbabwe (MoHCC). Operational and service delivery manual for the prevention, care and treatment of HIV in Zimbabwe. 2017. https://www.differentiatedcare.org/Portals/0/adam/Content/m2an155byU6RIoHeF4e4FQ/File/MSFZimOSDMwebrevised.pdf.

[64] Reif LK, Abrams EJ, Arpadi S, Elul B, McNairy ML, Fitzgerald DW (2020). Interventions to improve antiretroviral therapy adherence among adolescents and youth in low- and middle-income countries: a systematic review 2015–2019. AIDS Behav.

[65] World Medical Association (WMA). WMA declaration of Helsinki - ethical principles for scientific requirements and research protocols. Finland, Helsinki; 2018. https://www.wma.net/policies-post/wma-declaration-of-helsinki-ethical-principles-for-medical-research-involving-human-subjects.

[66] Council for International Organizations of Medical Sciences (CIOMS). International ethical guidelines for health-related research involving humans. Geneva, Switzerland; 2016. https://cioms.ch/wp-content/uploads/2017/01/WEB-CIOMS-EthicalGuidelines.pdf. 40523065

