# Supplementary material for: Virological outcomes and risk factors for non-suppression for routine and repeat viral load testing after enhanced adherence counselling during viral load testing scale-up in Zimbabwe: analytic cross-sectional study using laboratory data from 2014 to 2018
Source: AIDS Res Ther. 2022 Jul 9;19:34. doi: 10.1186/s12981-022-00458-z (PMC9270749; doi:10.1186/s12981-022-00458-z)
Supplement: Supplementary file 1 — Additional file 1: Table S1. Characteristic of patients who had repeat viral load tests between 2014 and 2015. Table S2. Association of exposure variables and viral load non-suppression for repeat tests between 2014 and 2018. Table S3. Bivariate and multivariate logistic regression analysis for repeat viral load tests done between 2014 and 2018. [file 12981_2022_458_MOESM1_ESM.docx]

**Additional Table 1: Characteristic of patients who had repeat viral load tests between 2014 and 2015*.***

| **Variable** | **Category** | **N=1884** | **Percentage (%)** |
| --- | --- | --- | --- |
| **Sex** | Female | 1141 | 60.6 |
|  | Male | 697 | 37.0 |
|  | Missing | 46 | 2.4 |
| **Median Age IQR* (Q1:Q3)** |  | 37(23-46) |  |
| **Age** | Children (0-9 years) | 120 | 6.4 |
|  | Adolescents (10-19years) | 270 | 14.3 |
|  | Young adults (20-24 years | 94 | 5.0 |
|  | Adults (25-49 years) | 1032 | 54.8 |
|  | Adults (≥ 50 years) | 324 | 17.2 |
|  | Missing | 44 | 2.3 |
| **ART Initiation Year** | <2010 | 652 | 34.6 |
|  | 2011-2013 | 775 | 41.1 |
|  | 2014-2015 | 409 | 21.7 |
|  | >2016 | 48 | 2.5 |
| **ART Regimen** | TDF based NNRTI | 1540 | 81.7 |
|  | non-based TDF NNRTI | 329 | 17.5 |
|  | PI Based | 15 | 0.8 |
|  | Other | 0 | 0.0 |
| **Pregnant** | Confirmed | 25 | 1.3 |
|  | Unconfirmed ** | 1859 | 98.7 |
| **Breastfeeding** | Confirmed | 80 | 4.2 |
|  | Unconfirmed** | 1804 | 95.8 |
| **Consent to receive VL results notification**  **via mobile text message reminder** | Yes | 1324 | 70.3 |
|  | No | 434 | 23.0 |
|  | Missing | 126 | 6.7 |
| **Level of Care** | Primary | 1297 | 68.8 |
|  | Secondary | 568 | 30.1 |
|  | Tertiary | 17 | 0.9 |
|  | Missing | 2 | 0.1 |
| **EAC sessions**  **performed.** | Yes | 374 | 19.9 |
|  | No | 428 | 22.7 |
|  | Missing | 1082 | 57.4 |
| **Number of EAC**  **sessions performed** | One | 37 | 9.9 |
|  | Two | 76 | 20.3 |
|  | Three | 180 | 48.1 |
|  | > Three | 57 | 15.2 |
|  | Missing | 24 | 6.4 |

**IQR: Interquartile range, TDF: Tenofovir, NNRTI: Non-nucleoside reverse transcriptase inhibitor*, *NRTI: Nucleoside reverse transcriptase inhibitor, PI: Protease Inhibitor, VL: viral load, EAC: enhanced adherence counselling*

***Unconfirmed includes males and females of all ages*

**Additional Table 2: Association of exposure variables and viral load non-suppression for repeat tests between 2014 and 2018.**

| **Variable** | **Category** | **Suppression** | | **Non-suppression** | | **p-value**** |
| --- | --- | --- | --- | --- | --- | --- |
|  |  | **n** | **%** | **n** | **%** |  |
| **Sex** | Female | 565 | 49.5 | 576 | 50.5 | **0.007** |
|  | Male | 297 | 42.6 | 400 | 57.4 |  |
|  | Missing | 26 | 56.5 | 20 | 43.5 |  |
| **Age** | Children (0-9 years) | 49 | 40.8 | 71 | 59.2 | **<0.001** |
|  | Adolescents (10-19 years) | 74 | 27.4 | 196 | 72.6 |  |
|  | Young adults (20-24 years) | 22 | 23.4 | 72 | 76.6 |  |
|  | Adults (25-49 years) | 531 | 51.5 | 501 | 48.5 |  |
|  | Adults(≥ 50 years) | 196 | 60.5 | 128 | 39.5 |  |
| **ART Initiation Year** | <2010 | 332 | 50.9 | 320 | 49.1 | 0.123 |
|  | 2011-2013 | 348 | 44.9 | 427 | 55.1 |  |
|  | 2014-2015 | 186 | 45.5 | 223 | 54.5 |  |
|  | >2016 | 22 | 45.8 | 26 | 54.2 |  |
| **Breastfeeding** | Confirmed | 42 | 52.5 | 38 | 47.5 | 0.326 |
|  | Unconfirmed* | 846 | 46.9 | 958 | 53.1 |  |
| **Pregnant** | Confirmed | 17 | 68.0 | 8 | 32.0 | **0.035** |
|  | Unconfirmed * | 871 | 46.9 | 988 | 53.1 |  |
| **Consent to receive VL results notification**  **via mobile text message reminder** | Yes | 649 | 49.0 | 62 | 51 | **0.005** |
|  | No | 175 | 40.3 | 259 | 59.7 |  |
|  | Missing | 64 | 50.8 | 62 | 49.5 |  |
| **Level of Care** | Primary | 600 | 46.3 | 697 | 53.6 | 0.30 |
|  | Secondary | 281 | 49.5 | 287 | 50.5 |  |
|  | Tertiary | 7 | 41.2 | 10 | 58.8 |  |
|  | Missing | 0 | 0.0 | 2 | 100 |  |
| **Unconfirmed includes males and females of all ages*  ***Chi-square* | | | | | | |

**Additional Table 3: Bivariate and multivariate logistic regression analysis for repeat viral load tests done between 2014 and 2018.**

| **Variables** | **Categories** | **OR** | **p-value** | **(95%CI)** | **aOR** | **p-value** | **(95%CI)** |
| --- | --- | --- | --- | --- | --- | --- | --- |
| **Sex** | Female | 1 | **0.007** |  |  |  |  |
|  | Male | 1.32 |  | (1.09-1.60) |  |  |  |
|  | Missing | 0.75 |  | (0.41-1.36) |  |  |  |
| **Age** | Adults (25-49 years) | 1 | **<0.001** |  |  | **<0.001** |  |
|  | Children (0-9 years) | 1.54 |  | (1.05-2.26) | 1.51 |  | (1.03-2.22) |
|  | Adolescents (10-19 years) | 2.81 |  | (2.10-3.78) | 2.76 |  | (2.11-3.72) |
|  | Young adults (20-24 years) | 3.47 |  | (2.15-5.80) | 3.48 |  | (2.16-5.83) |
|  | Adults (≥50 years) | 0.69 |  | (0.54-0.89) | 0.68 |  | (0.53-0.88) |
| **Pregnant** | Unconfirmed* | 1 | **0.034** |  |  | **0.05** |  |
|  | Confirmed | 0.41 |  | (0.17-0.94) | 0.44 |  | (0.18-1.00) |
| **Consent to receive VL results notification**  **via mobile text message reminder** | No | 1 | **0.005** |  |  |  |  |
|  | Yes | 0.70 |  | (0.56-0.87) |  |  |  |
|  | Missing | 0.65 |  | (0.44-0.98) |  |  |  |
| **Unconfirmed includes males and females of all ages* | | | | | | | |
